# TNF-α mediated upregulation of Na_V_1.7 currents in rat dorsal root ganglion neurons is independent of CRMP2 SUMOylation

**DOI:** 10.1186/s13041-019-0538-0

**Published:** 2019-12-30

**Authors:** Flávio Henrique Pequeno de Macedo, Rosária Dias Aires, Esdras Guedes Fonseca, Renata Cristina Mendes Ferreira, Daniel Portela Dias Machado, Lina Chen, Fang-Xiong Zhang, Ivana A. Souza, Virgínia Soares Lemos, Thiago Roberto Lima Romero, Aubin Moutal, Rajesh Khanna, Gerald W. Zamponi, Jader S. Cruz

**Affiliations:** 1 Department of Biochemistry and Immunology, Federal University of Minas Gerais, Belo Horizonte, Brazil; 2 Department of Physiology and Pharmacology, Hotchkiss Brain Institute and Alberta Children’s Hospital research Institute, University of Calgary, Calgary, Canada; 3 Department of Pharmacology, University of Arizona, Tucson, AZ USA

**Keywords:** Diabetic neuropathic pain, Tumor necrosis factor, DRG neurons, Sodium channel Na_V_1.7

## Abstract

Clinical and preclinical studies have shown that patients with Diabetic Neuropathy Pain (DNP) present with increased tumor necrosis factor alpha (TNF-α) serum concentration, whereas studies with diabetic animals have shown that TNF-α induces an increase in Na_V_1.7 sodium channel expression. This is expected to result in sensitization of nociceptor neuron terminals, and therefore the development of DNP. For further study of this mechanism, dissociated dorsal root ganglion (DRG) neurons were exposed to TNF-α for 6 h, at a concentration equivalent to that measured in STZ-induced diabetic rats that developed hyperalgesia. Tetrodotoxin sensitive (TTXs), resistant (TTXr) and total sodium current was studied in these DRG neurons. Total sodium current was also studied in DRG neurons expressing the collapsin response mediator protein 2 (CRMP2) SUMO-incompetent mutant protein (CRMP2-K374A), which causes a significant reduction in Na_V_1.7 membrane cell expression levels. Our results show that TNF-α exposure increased the density of the total, TTXs and TTXr sodium current in DRG neurons. Furthermore, TNF-α shifted the steady state activation and inactivation curves of the total and TTXs sodium current. DRG neurons expressing the CRMP2-K374A mutant also exhibited total sodium current increases after exposure to TNF-α, indicating that these effects were independent of SUMOylation of CRMP2. In conclusion, TNF-α sensitizes DRG neurons via augmentation of whole cell sodium current. This may underlie the pronociceptive effects of TNF-α and suggests a molecular mechanism responsible for pain hypersensitivity in diabetic neuropathy patients.

## Introduction

The World Health Organization (WHO) defines diabetes as a chronic disease that results from poor insulin production or the inability of the body to use it efficiently. As a result, basal glucose concentration in the bloodstream rises, resulting in hyperglycemia [1] According to estimates, 9% of the world’s population over 18 years old are affected by diabetes [2], while around 1.6 million deaths were caused directly by diabetes, in 2016 [3]. The WHO projection points out that, by 2030, diabetes will be the 7th major cause of death in the world [4]. Patients with diabetes suffer from macrovascular complications, such as myocardial infarction, stroke, peripheral vascular disease, microvascular complications that manifest as peripheral neuropathy, retinopathy and nephropathy [5]. Diabetes is the main cause of peripheral neuropathy [6]. Among the various types of diabetic neuropathy, the most common clinical manifestation is distal symmetric polyneuropathy, also called peripheral diabetic neuropathy (PDN), which affects 75% of patients with diabetic neuropathy [7]. Approximately 20 to 30% of patients with PDN suffer from diabetic neuropathic pain (DPN), one of the main clinical consequences of PDN [8, 9].

Studies in Streptozotocin (STZ)-induced diabetic rats show that the development of hyperalgesia presented by these animals is associated with an increased expression of voltage-dependent sodium (Na_V_) channels [10, 11]. In addition, patch clamp recordings indicated increased TTX-sensitive sodium current density consistent with increased expression of the Na_V_1.3, 1.6 and 1.7 isoforms [12–14]. Among these, the Na_V_1.7 isoform has been directly linked to diabetic neuropathy and the release of proinflammatory cytokines [11, 15–17]. These channels are mostly expressed in small diameter Aδ and C fibers [18] and, not surprisingly, in 85% of functionally identified nociceptors [19]. In addition, Na_V_1.7 expression is increased in DRG neurons of STZ-induced diabetic rats, a change that contributes to pain-related hypersensitivity [11, 16, 20]. In PDN, increased tumor necrosis factor α (TNF-α) expression in dorsal root ganglion (DRG) neurons was linked to increased Na_v_ 1.7 levels and nociceptive behaviors.

Here, we first determined the TNF-α serum concentration in a rat model of PDN and then assessed, for the first time, the effects of this specific and physiologically relevant concentration on whole cell sodium currents in DRG neurons from normoglycemic rats. Then, we investigated whether manipulating a recently reported Na_V_1.7 trafficking regulator, the collapsin response mediator protein 2 (CRMP2) [21], could normalize the TNF-α mediated increase in Na_V_1.7 function. CRMP2 is a cytosolic phosphoprotein that is dysregulated in neuropathic pain [8, 22]. Its function as a trafficking regulator for Na_V_1.7 [21, 23, 24] was suggested to be instrumental in regulating allodynic and hyperalgesic behaviors in various rodent models of pain [5, 6]. When SUMOylated, CRMP2 protects Na_V_1.7 from endocytosis thus maintaining the availability of the channel for voltage-dependent activation and nociceptive transmission [22, 23]. Inhibition of CRMP2 SUMOylation efficiently decreased Na_V_1.7 surface localization and currents [25]. Thus, we hypothesized that inhibiting CRMP2 SUMOylation might reverse the increased Na_V_1.7 currents induced by TNF-α. However, as we show here, although TNF-α increases sodium current density, these effects occur independently of CRMP2 SUMOylation.

## Materials and methods

Experiments were approved by the Institutional Animal Care and Use Committee from the Federal University of Minas Gerais (protocol number 233/2013) and by the Health Sciences Animal Care Committee, from the University of Calgary (protocol number #AC13–0045).

### Induction of experimental diabetes

28 day old male Wistar rats were randomly assigned to both the diabetic and control groups. Rats in the diabetic group were fasted overnight before receiving a single intraperitoneal injection of STZ solution (65 mg/kg diluted in a 10 mM sodium citrate buffer solution, pH of 4.5) [26]. Rats in the control group received only sodium citrate buffer solution. As shown by Junod et al. [27], this single STZ dose induces a severe hypoglycemic state within 7 hours, and this matches the time course of development of hyperinsulinemia. Following STZ injection, waterers containing a 10% glucose solution were placed in the rat cages to reduce or prevent hyperinsulinemia and hypoglycemic shock [28]. Glycemia levels were assessed using blood samples (Accu-Check Active®, Roche) obtained immediately before STZ injection (day 0), and then monitored fortnightly until the day 60 after diabetes induction. Animals with blood glucose levels over 300 mg/dl were considered diabetic.

### Evaluation of mechanical hyperalgesia

Mechanical hyperalgesia was assessed by the use of a Randall–Selitto device (Ugo-basile, 37,215, Verase, Italy), through which an increasing pressure (32 g/s) was applied to the dorsal portion of the rats’ back paws. The force (g) that led the rat to withdraw its paw was considered as the mechanical nociceptive threshold. The increasing pressure cutoff was set at 250 g to prevent tissue damage [22].

### Quantification of TNF-α by ELISA

At the day before and at day 60 after STZ injection, 0.5 to 1.5 ml of blood samples were collected from the rats’ arterial tails by using a peripheral intravascular catheter (24G needle) perfused with sodium citrate (1 mM) to avoid coagulation during blood collection. Samples were kept at room temperature for 10 min, followed by refrigeration (4 °C) for 10 min to form the clot to obtain serum blood. The samples were then centrifuged (1300 RPM, 5 min) and the supernatant was collected. Plasma concentration of TNF-α was quantified with rat-specific ELISA kits (DuoSet kits; R&D Systems) using the Thermoscientific Multiskan FC. Kits were used in accordance with manufacturer’s instructions.

### DRG neuron dissociation

Sprague-Dawley rats (5–6 weeks old) were anesthetized with isoflurane and decapitated by the use of guillotine. After access to the spinal cord, 40–50 DRGs were aseptically dissected from cervical, thoracic and lumbar spinal segments, and kept in ice-cold PBS (in mM: 137 NaCl_2_, 2.7 KCl, 10 Na_2_HPO_4_ and 2 KH_2_PO_4_). After cleaning, DRGs were subjected to enzymatic digestion. To that end, they were exposed to 1 ml of F12 culture medium solution (supplemented with 10% v/v fetal bovine serum and 1% v/v penicillin-streptomycin solution) with the addition of 40 μl of papain and 4 mg of collagenase type 1 for 30 min (Invitrogen), kept in a water bath at 37 °C. The DRGs were slightly shaken every 5 min. Then the medium was replaced for the addition of 0.4% type IV DNAse (Sigma). The DRGs were kept for another 10 min in the water bath. After digestion, the culture medium was replaced 3 times in order to eliminate the digestive enzymes. DRGs were cautiously thinned by the use of a 1000 μl auto-pipette and the neurons were transferred to a supplemented F12 culture medium (B27 (2%), L-glutamine (1%), N2 (1%) and NGF (0.1%), Gibco). The dissociated DRG neurons were then distributed into a 24-well plate containing coverslips pretreated with laminin (1%) and Poly-D-lysine (10%). 600 μl of medium containing cells was added to each well. The 24-well plate was kept in the incubator (5% CO_2_–95% O_2_, 37 °C) until the cells were used. For the study of the TNF-α effect on the total sodium current in transfected DRG neurons, the cells were kept in culture for a period of 2 weeks. For the study of the effect of TNF-α on the TTXs and TTXr sodium currents, cells were used after a minimum time period of 12 h in culture.

### Virus production for infection of dissociated DRG neurons

For the infection of the dissociated DRG neurons, the AAV5 recombinant adenovirus (AAV5 Helper-Free System, Agilent Technologies Stratagene Products Division, CA, USA) kit was used. The kit contains three vectors: pAAV-MSC, pAAV-RC5 and pHelper. The first vector contained the genes of interest, CRMP2-WT and CRMP2-K374A, both tagged with GFP (Green Fluorescent Protein). These constructs were transfected into immortalized cells of the 293AAV line (by the use of calcium phosphate buffer solution) concomitantly with the vectors pAAV-RC5 and pHelper, a process that results in the production of viruses specific for infection of DRG neurons and containing the constructs of interest, CRMP2-WT-GFP and CRMP2-K374A-GFP, as viral materials. 24–72 h post-transfection, the viruses were extracted from the 293AAV cells accordingly with the instructions of a virus purification kit (Takara AAVpro® Purification Kit, TAKARA BIO INC), resulting in solutions containing 5∙10^7^ virus Gc/μl (genomic copies per microliter).

### DRG neuron infection

12–18 h after dissociation of the DRG neurons, 5 μl of solution containing the GFP-CRMP2-WT virus or 10 μl of solution containing the GFP-CRMP2-K374A virus was added to each well. Viruses were maintained in the culture for 72 h, after which the medium was replaced for a B27 (2%), L-glutamine (1%), N2 (1%) and NGF supplemented F12 medium. After 2 weeks in culture, successfully infected neurons emitted GFP fluorescence (Zeiss LSM-510, λ = 488 nm), confirming that they expressed the plasmid.

For the study of the TNF-α exposure effect on the total Na^+^ current in infected cells, TNF-α (TNF Recombinant Rat Protein, Thermo Fisher Scientific) was added to the culture medium at a concentration of 700 pg/ml for 6 h, after which the cells had measured their total Na^+^ current in a Patch Clamp platform.

### Electrophysiology

The total Na^+^ current was recorded by using the amplifier Axoclamp 200B in the whole cell voltage clamp configuration in combination with the Clampex 9.2 software (Molecular Devices, Sunnyvale, CA). Low resistance patch electrodes (3–4 MΩ) were filled with solution containing (in mM): 10 NaCl, 100 CsCl, 5 MgCl_2_, 10 HEPES and 11 EGTA and 10 TEA-Cl, pH 7.2 adjusted with 1 M CsOH. 10 NaCl, 100 CsCl, 5 MgCl_2_, 10 HEPES and 11 EGTA and 10 TEA-Cl, pH 7.2 adjusted with 1 M CsOH. Cells were initially kept in a bath solution containing (in mM): 50 NaCl, 5 CsCl, 0.1 CdCl_2_, 0.5 MgCl_2_, 60 Glucose and 5 HEPES, pH 7.4 adjusted with 1 M NaOH. After reaching the whole cell configuration, the cell was perfused with external solution containing (in mM): 40 NaCl, 3 KCl, 1 CaCl_2_, 1 MgCl_2_, 0.1 CdCl_2_, 20 TEA-Cl, 70 Choline-Cl, 10 HEPES and 10 Glucose, pH 7.4 adjusted with 1 M HCl/NaOH. Liquid junction potentials between internal and bath solutions (− 0.5 mV) and between internal and external solutions (4.8 mV) were corrected before any recordings. An Ag-AgCl electrode was used as reference. The recordings were filtered with a Bessel lowpass filter set at 2.9 kHz and digitalized at a 20 kHz (50 μs interval) through a Digidata 1320A interface board. Capacitive currents were electronically compensated and a P/4 protocol was used for correction of the linear leakage current and for the subtraction of the residual capacitance [24]. The experiments were carried out on a petri acrylic plate, 35 mm in diameter, using an inverted microscope (Nikon TMF- 100, Nikon, Japan).

For patch clamp experiments involving the acute effect of TNF-α exposure, Na^+^ current recordings were obtained by using the Patch Clamp amplifiers type EPC-9/EPC-10 (HEKA Instruments, Germany) and the PULSE/PATCHMASTER data acquisition program (HEKA Instruments, Germany) adjusted for the whole cell voltage clamp configuration. Low resistance patch electrodes (3–4 MΩ) were filled with the same pipette solution mentioned before, as well as the bath/external solution. An Ag- AgCl was used as a reference. Capacitive currents were electronically compensated and a P/4 protocol was used to correct the linear leakage current and to subtract residual capacity [24]. The current recordings were filtered with a Bessel lowpass filter set at 2.9 kHz and acquired at a rate of 20 kHz (50 μs interval) through an AD/DA interface (ITC 1600). The experiments were performed on 35 mm diameter acrylic Petri dishes using inverted microscope (Axiovert 20, Carl Zeiss, Germany or Nikon TMF-100, Nikon, Japan). To record the TTXr current, after establishing the whole cell configuration and obtaining the total Na^+^ current, 100 μl of TTX-containing external solution was added to the bath solution to give a final TTX concentration of 300 nM. Data were acquired 20 s after TTX was added.

### Data analyses

The Na^+^ current was recorded from neurons with capacitance ≤45 pF (diameters between 15 and 30 μm) [11, 29, 30]. Current voltage (I-V) relations were fitted with the equation

1 $$ I\left({V}_m\right)=\frac{G_{max}\cdot \left({V}_m-{V}_r\right)}{1+{e}^{\left({V}_{1/2}-{V}_m\right)/k}} $$

where I (V_m_) is the current for a given membrane potential (V_m_), V_r_ is the reversal potential, G_max_ is the maximum conductance, V_1/2_ is the half activation potential and κ is the slope factor. The normalized conductance was obtained by the G/G_max_ ratio. Steady state inactivation curves were fitted with the equation

2 $$ {h}_{\infty }=\frac{1}{1+{e}^{\left({V}_m-{V}_h\right)/{k}_h}} $$

where V_h_ is the half inactivation potential and κ_h_ is the slope of the steady state inactivation curve. The window current probability graph was obtained by the product between the equations for the steady state activation and the steady state inactivation curve [31], as described in Eq. 3.

3 $$ p=\frac{1}{1+{e}^{\left({V}_{1/2}-{V}_m\right)/k}}\cdot \frac{1}{1+{e}^{\left({V}_m-{V}_h\right)/{k}_h}} $$

TTXs Na^+^ currents were isolated by digital subtraction between total Na^+^ current and TTXr Na^+^ current, the latter obtained by the use of TTX.

### Statistics

One- and two-way analysis of variance followed by Bonferroni tests was used for multiple comparisons as stated in the figure legends. Statistical significance was set at 0.05.

## Results

### Diabetic rats develop hyperalgesia and increasing in TNF-α serum concentration

**Fig. 1:**
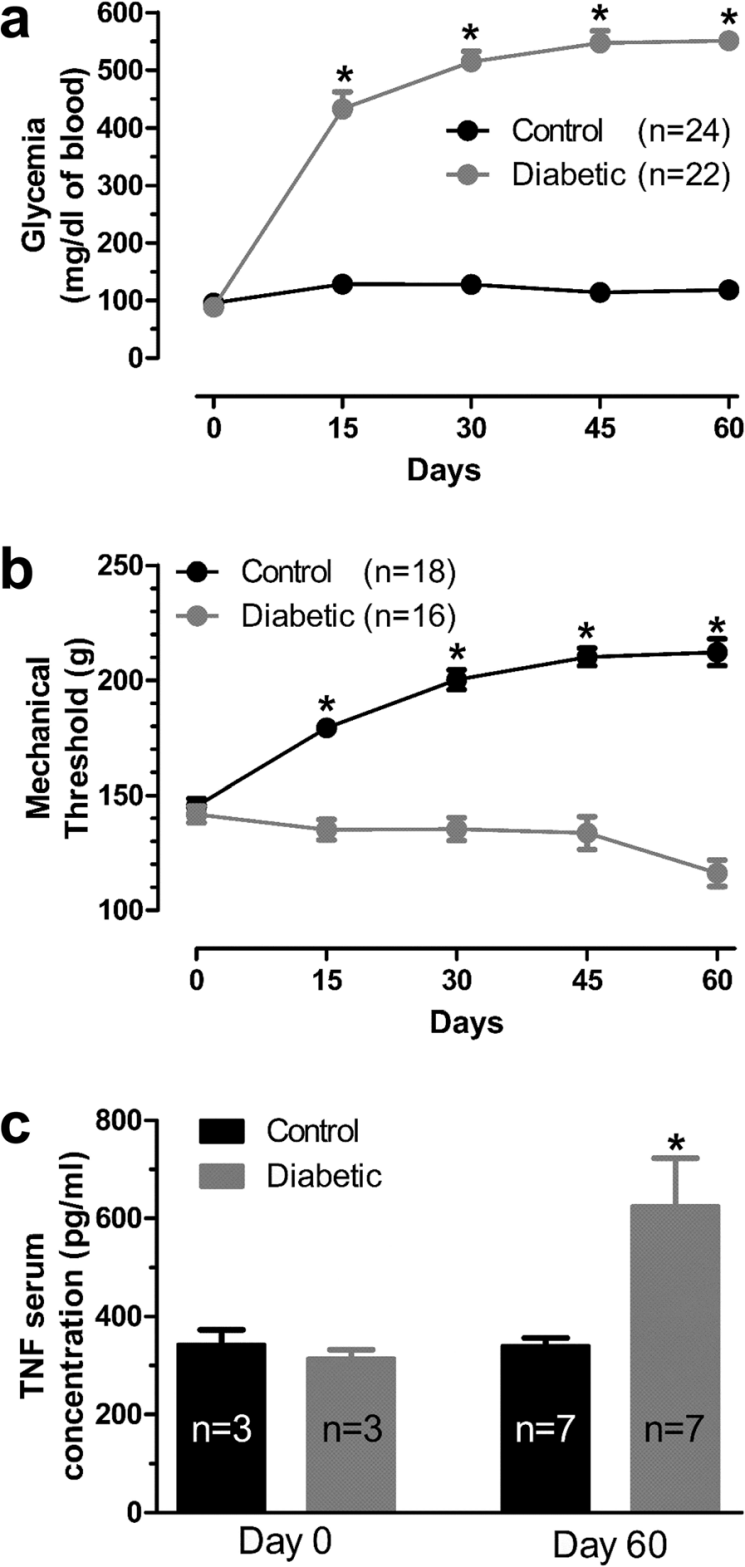
Glucose level and behavioral analysis in diabetic rats. **a** Glycemia values measured biweekly. n represents the number of rats * *p* < 0.05 control vs diabetic; Two Way ANOVA test followed by Bonferroni. **b** Mechanical thresholds measured biweekly. * *p* < 0.05 control vs diabetic. Two Way ANOVA test followed by Bonferroni. **c** TNF-α serum concentration dosage in the Control and Diabetic group for both day 0 and 60. n reflects numbers of rats. * *p* < 0.05 control vs diabetic; One Way ANOVA test followed by Bonferroni

Induction of diabetes by intraperitoneal (i.p.) STZ injection resulted in sustained hyperglycemia of diabetic rats for at least 60 days (Fig. 1a and Table 1). Concomitantly, the mechanical withdrawal thresholds of diabetic rats progressively decreased over the course of 60 days, whereas those of the control group showed a progressive increase over this time period, altogether indicating behavioral sensitization of the diabetic group (Fig. 1b and Table 2). We also assessed the TNF-α serum concentration of STZ treated and control rats. On day 60, the diabetic rats showed an elevation in TNF-α serum concentration level (Control, 340.3 ± 16.0 pg/ml vs Diabetic, 624.9 ± 97.8 pg/ml, day 60, Fig. 1c and Table 3**)**.

**Table 1:** Comparison of glycemia levels (mg/dl of blood) between Control and Diabetic rats

	n	day 0	day 15	day 30	day 45	day 60
Control	24	95.4 ± 4.2	127.8 ± 2.0	127.5 ± 2.0	113.8 ± 1.8	117.9 ± 1.7
Diabetic	22	87.8 ± 3.4	432.8 ± 29.3*	514.0 ± 18.3*	546.7 ± 21.2*	550.7 ± 10.6*
*p* value		0.6687	< 0.001	< 0.001	< 0.001	< 0.001

*Significant by Two Way ANOVA test, followed by Bonferroni (control vs diabetic)

**Table 2:** Comparison of mechanical thresholds (g) between Control and Diabetic rats

	day 0	day 15	day 30	day 45	day 60
Control	145.4 ± 3.3 *n* = 19	179.3 ± 2.5 *n* = 19	200.2 ± 4.3 *n* = 18	210.1 ± 3.8 *n* = 18	212.1 ± 5.9 *n* = 18
Diabetic	141.8 ± 3.6 *n* = 29	135.1 ± 4.5* *n* = 28	135.3 ± 4.5* *n* = 18	133.7 ± 7.2* *n* = 18	116.1 ± 5.8* *n* = 16
p value	0.5555	< 0.001	< 0.001	< 0.001	< 0.001

*Significant by Two Way ANOVA test, followed by Bonferroni (control vs diabetic)

**Table 3:** Comparison of TNF-α serum concentration (pg/ml) between Control and Diabetic rats

	day 0	day 60
Control	343.0 ± 30.3 *n* = 3	340.3 ± 15.9 *n* = 7
Diabetic	314.2 ± 18.2 *n* = 3	624.9 ± 97.8* *n* = 7
p value	0.9031	0.0109

*Significant by One Way ANOVA test, followed by Bonferroni (control vs diabetic)

### TNF-α induces modulation of DRG neurons Na^+^ channels

**Fig. 2:**
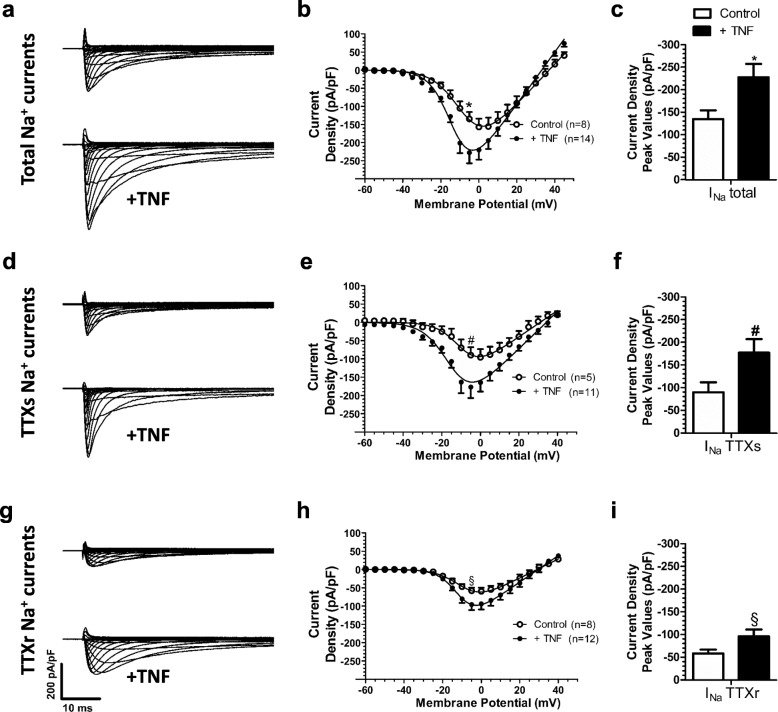
Effects of TNF-α exposure on sodium current amplitude in DRG neurons Representative trace of the total sodium current recorded from cells with and without exposure to TNFα (**a**)**,** along with their normalized current density-voltage relations (**b**). **c** Peak current density values for the total sodium current with and without exposure to TNF-α. **d, e, f** same as in panels **a-c** but for the TTXs sodium current component. **g, h, i** same as in panels **a-c** but for the TTXr sodium current component. For panels **c, f** and **i**, peak currents were measured at − 5 mV. * Total sodium current vs total sodium current after exposure to TNF-α, # TTXs sodium current vs TTXs sodium current after exposure to TNF, § TTXr sodium current vs TTXr sodium current after TNF exposure - *p* < 0.05; One Way ANOVA test followed by Bonferroni. For panels **b, e** and **h**, * Total sodium current vs total sodium current after exposure to TNF-α, # TTXs sodium current vs TTXs sodium current after exposure to TNF, § TTXr sodium current vs TTXr sodium current after TNF exposure - *p* < 0.05; Two Way ANOVA test followed by Bonferroni

**Fig. 3:**
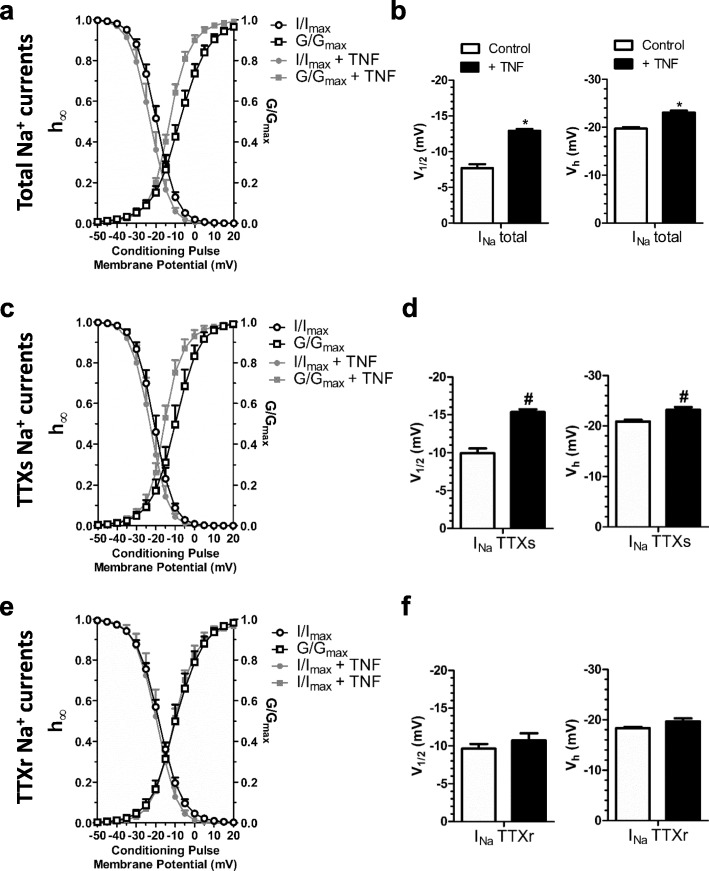
Effects of TNF-α exposure on sodium current gating in DRG neurons (**e**) Steady state activation and inactivation curves for the total sodium current recorded from cells with and without TNF-α exposure (**b**) Comparison of the half-activation and half-inactivation potential for total sodium current. **c, d** same as in panels **a** and **b**, but for the TTXs sodium current component. **e, f** same as in panels **a** and **b**, but for the TTXr sodium current component. * Total sodium current vs total sodium current after exposure to TNF-α, # TTXs sodium current vs TTXs sodium current after exposure to TNF, § TTXr sodium current vs TTXr sodium current after TNF exposure - *p* < 0.05; One Way ANOVA test followed by Bonferroni

As shown by Tamura et al. [16], adrenal chromaffin cells achieved maximum Nav1.7 protein expression after 6 hour exposure to 100 ng/ml TNF-α. Based on the findings of Fig. 1c and Table 3, the physiologically relevant TNF-α concentration (700 pg/ml) was added to the medium of dissociated DRG neurons for 6 hours. After 6 h of TNF-α exposure, the total Na^+^ current, as well as its TTXs and TTXr currents components were determined **(**Fig. 2**)**. TNF-α induced an increase in the total Na^+^ current density in DRG neurons, as well as that of both the TTXs and TTXr components (Fig. 2c, f, j, and Table 4). In addition, total Na^+^ and TTXs currents appeared to activate at more negative voltages after TNF-α exposure, as evident from a leftward shift in the IV relation **(**Fig. 2b, e, h**)** and corresponding steady state activation curves (Fig. 3a, c, e, and Table 5), leading to a significantly more hyperpolarized half-activation voltage (Fig. b, d, f, and Table 6).

**Table 4:** Comparison of the peak current density values (pA/pF) at a test potential of − 5 mV between the INa Total, INa TTXs and Ina TTXr with and without TNF-α

	I_Na_ Total	I_Na_ TTXs	I_Na_ TTXr	
Control	− 134.7 ± 30.3 *n* = 8	−89.7 ± 21.9 *n* = 5	− 58.2 ± 8.4 *n* = 8	
+ TNF	− 227.9 ± 29.2* *n* = 14	− 177.0 ± 29.8* *n* = 11	−95.9 ± 14.7* *n* = 12	
p value	< 0.001	0.0027	0.0044	

*Significant by One Way ANOVA test, followed by Bonferroni (control vs TNF)

**Table 5:** Comparison of the slope factor (k) values (mV) for steady state activation and steady state inactivation curves with and without TNF-α

	steady state activation k factor			steady state inactivation k factor		
	I_Na_ Total	I_Na_ TTXs	I_Na_ TTXr	I_Na_ Total	I_Na_ TTXs	I_Na_ TTXr
Control	7.2 ± 0.4 *n* = 8	6.3 ± 0.5 *n* = 6	6.8 ± 0.5 *n* = 8	− 5.1 ± 0.2 *n* = 7	−4.8 ± 0.2 *n* = 6	−5.9 ± 0.1 *n* = 6
+ TNF	5.3 ± 0.2* *n* = 14	4.8 ± 0.3* *n* = 11	5.3 ± 0.8 *n* = 15	−5.0 ± 0.3 *n* = 6	−4.7 ± 0.4 *n* = 4	−5.2 ± 0.5 *n* = 4
p value	< 0.001	0.0012	0.5598	0.3861	0.7267	0.2197

*Significant by One Way ANOVA test, followed by Bonferroni (control vs TNF)

**Table 6:** Comparison of the V_50_ and V_h_ values (mV) for steady state activation and steady state inactivation curves with and without TNF-α

	V_1/2_			V_h_		
	I_Na_ Total	I_Na_ TTXs	I_Na_ TTXr	I_Na_ Total	I_Na_ TTXs	I_Na_ TTXr
Control	−7.7 ± 0.5 *n* = 8	−9.9 ± 0.6 *n* = 6	− 9.6 ± 0.6 *n* = 8	−19.7 ± 0.2 *n* = 7	−20.8 ± 0.3 *n* = 6	−18.3 ± 0.2 *n* = 6
+ TNF	−12.9 ± 0.2* *n* = 14	−15.3 ± 0.3* *n* = 11	−10.7 ± 0.9 *n* = 15	−23.0 ± 0.4* *n* = 6	−23.1 ± 0.5* *n* = 4	− 19.6 ± 0.5 *n* = 4
p value	< 0.001	< 0.001	0.2589	< 0.001	0.0052	0.4003

*Significant by One Way ANOVA test, followed by Bonferroni (control vs TNF)

**Fig. 4:**
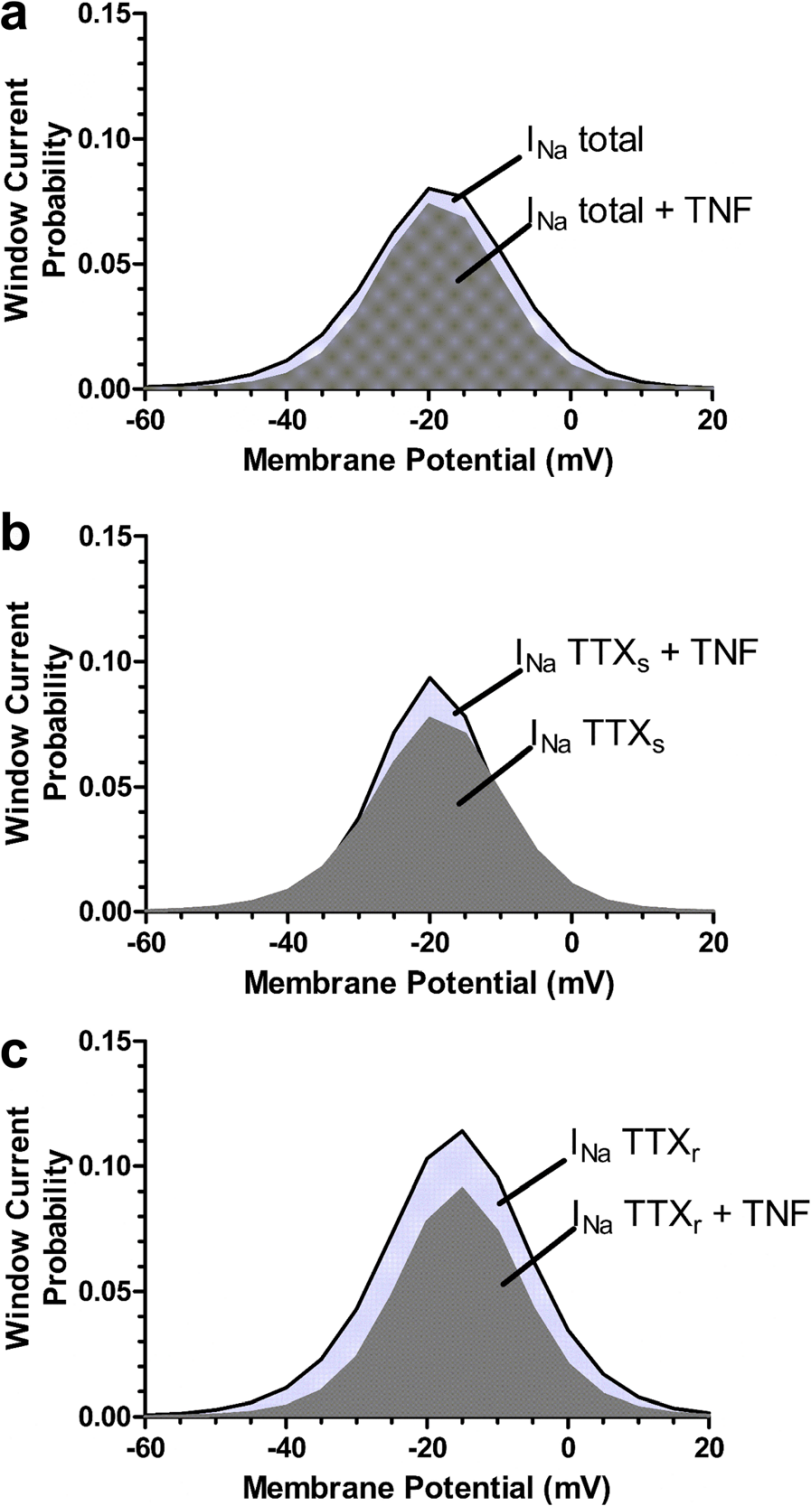
Analysis of window currents*.* Window current probability obtained from the activation and inactivation curves depicted in Fig. 2 for the effect of TNF-α exposure on (**a**) the total sodium window current, (**b**) the TTXs sodium window current and (**c**) the TTXr sodium current vs TTXr sodium window current

An analysis of steady state inactivation properties showed that the TTXs and TTXr currents respond differently to TNF-α exposure. There was a leftward shift in the steady state inactivation curves for the total Na^+^ and TTXs currents, leading to a more hyperpolarized half-inactivation potential **(**Fig. 3a, c, e and Fig. 3b, d, f) that was not observed with TTXr currents. A calculation of the product of the activation and steady-state inactivation relationships reveals the impact of the TNF-α induced shifts on sodium window current (Fig. 4). TNF-α treatment slightly reduced total Na^+^ window current probability **(**Fig. 4a). Analyzing TTXs (Fig. 4b) and TTXr **(**Fig. 4c**)** components revealed that TNF-α treatment promoted both an increase in the peak and a slight leftward shift of the TTXs window current probability, whereas a reduction in TTXr window current probability is observed.

Altogether, these data indicate that a concentration of TNF-α equivalent to that seen in diabetic produces a gain of function predominantly in TTXs sodium currents of DRG neurons.

### CRMP2 SUMOylation is not involved in TNF-α mediated increases in Na_V_1.7 membrane expression

**Fig. 5:**
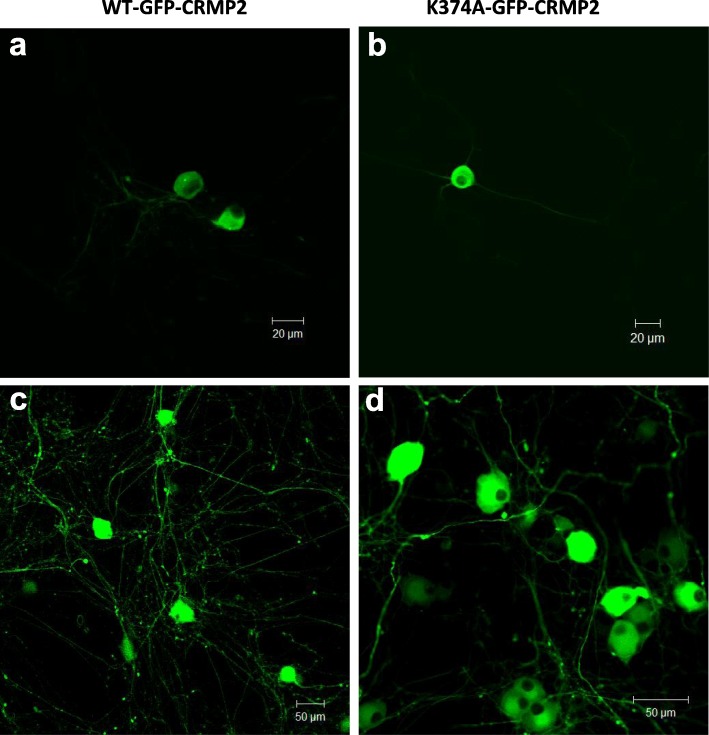
Confocal microscopy images taken from dissociated DRG neurons 2 weeks after viral infection (**a**) Examples of DRG neurons expressing the CRMP2-WT tagged with GFP. **b** DRG neurons expressing the CRMP2-K374A with a GFP tag. **c** and **d**. Images of axons arising from DRG neuron cells bodies expressing CRMP2-WT-GFP and CRMP2-K374A-GFP, respectively

**Fig. 6:**
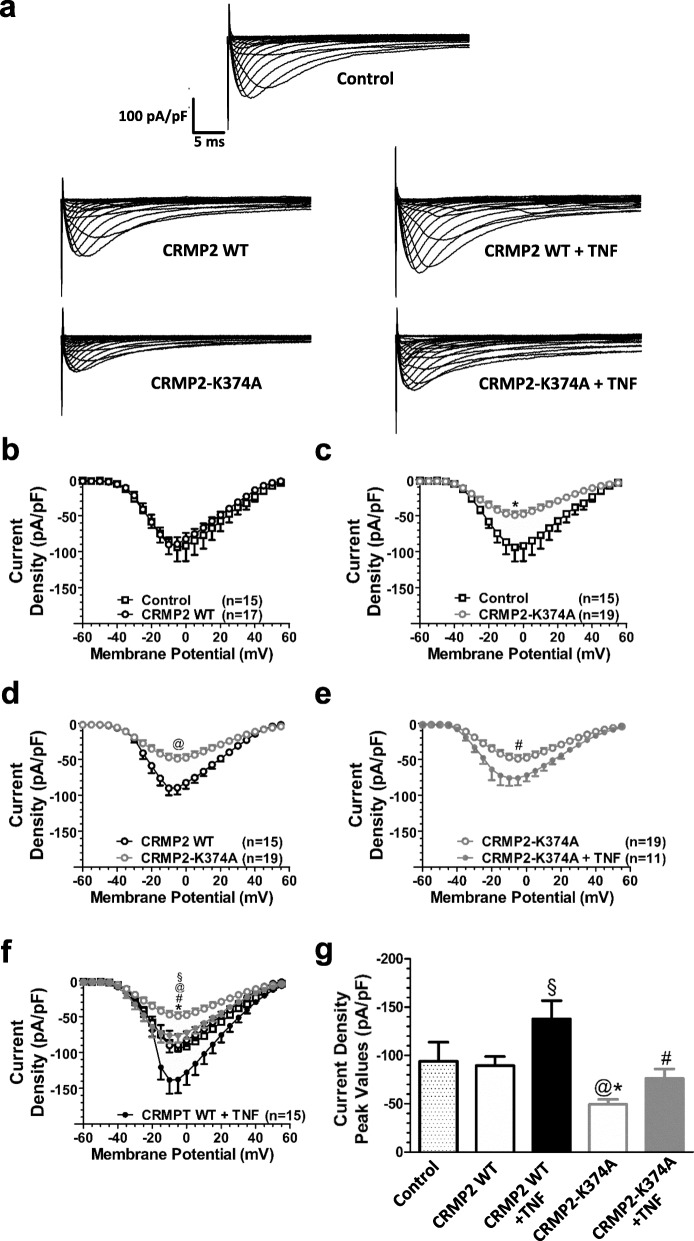
Sodium currents in DRG neurons expressing CRMP2 and its mutants*.* **a** Representative traces recorded from a non-transfected DRG neuron (control), of the total sodium current recorded from DRG neurons expressing CRMP2-WT-GFP or CRMP2-K374A-GFP without exposure to TNF-α and after being exposed to TNFα for 6 h. **b-f** comparisons of the current density-voltage relationships for the total sodium current recorded from control, CRMP2-WT and CRMP2-K374A neurons. For panels (**b**) and (**c**), the control group is compared to CRMP2-WT and CRMP2-K374A groups, respectively. For panels (**d**) and (**e**), the CRMP2-K374A group is compared to CRMP2-WT and CRMP2-K374A + TNF-α groups, respectively. For panel (**f**), all previous groups are depicted, and the CRMP2-WT + TNFα group is added. § control vs CRMP2 WT + TNFα; * control vs CRMP2-K374A; @ CRMP2 WT vs CRMP2-K374A; # CRMP2-K374A vs CRMP2-K374A + TNFα. §, *, @ and #, *p* < 0.05; Two Way ANOVA test followed by Bonferroni. **g** shows the current density peak values measured at − 5 mV for all of the studied groups. *, # and §, *p* < 0.05; One Way ANOVA test followed by Bonferroni. n reflects numbers of cells. + TNF reflects 6 h TNF-α exposure

Previous work has shown that CRMP2, in its SUMOylated form, is a potent regulator of Na^+^ channel membrane localization in the primary afferent pain pathway [21–24]. To determine whether TNF-α acts through this pathway, the total Na^+^ current was recorded from dissociated DRG neurons infected with AAV5 constructs encoding a CRMP2 SUMO-incompetent mutant protein, CRMP2-K374A-GFP. Control cells were infected with wild type CRMP2-GFP-AAV5 (Fig. 5). After being kept in culture for 2 weeks, the infected DRG neurons exhibited strong expression of the various CRMP2-GFP constructs (Fig. 5a-d) and robust Na_V_ currents (Fig. 6a). DRG neurons infected with CRMP2-WT exhibited total Na^+^ current density values (− 89.4 ± 9.3 pA/pF) similar to that obtained in non-infected (control) DRG neurons (− 94 ± 19.6 pA/pF, Fig. 6b, g, and Table 7). DRG neurons expressing CRMP2-K374A showed reduced total Na^+^ current density (− 49.2 ± 5.3 pA/pF) when compared to both the control cells and CRMP2-WT cells (Fig. 6c, d, g and Table 7). After exposure to TNF-α, CRMP2-WT expressing cells showed a 40% increase in total Na^+^ current density (− 137.6 ± 19 pA/pF) (Fig. 6f, g and Table 7). TNF-α treatment increased total Na^+^ current density in CRMP2-K374A expressing cells by about 50% (− 76 ± 9.9 pA/pF) (Fig. 6e, g and Table 7). Hence, we conclude that interfering with CRMP2 SUMOylation does not preclude TNF-α mediated increases in Na^+^ current density.

**Table 7:** Comparison of peak current density (pA/pF), at a test potential of − 5 mV, recorded from Control cells and cells expressing various CRMP2 constructs with and without exposure to TNF-α

Control	CRMP2 WT	CRMP2-K374A	CRMP2 WT + TNF-α	CRMP2-K374A + TNF-α	p value
−93.6 ± 19.6 *n* = 15	− 89.4 ± 9.2 *n* = 17	–	–	–	0.6722
−93.6 ± 19.6 *n* = 16	–	− 49.2 ± 5.2* *n* = 19	–	–	< 0.001
−93.6 ± 19.6 *n* = 17	–	–	− 137.6 ± 18.9* *n* = 15	–	< 0.001
−93.6 ± 19.6 *n* = 18	–	–	–	− 76.0 ± 9.8 *n* = 11	0.1420
–	− 89.4 ± 9.2 *n* = 17	−49.2 ± 5.2* *n* = 19	–	–	< 0.001
–	− 89.4 ± 9.2 *n* = 17	–	− 137.6 ± 18.9* *n* = 15	–	< 0.001
–	− 89.4 ± 9.2 *n* = 17	–	–	− 76.0 ± 9.8 *n* = 11	0.2566
–	–	− 49.2 ± 5.2 *n* = 19	−137.6 ± 18.9* *n* = 15	–	< 0.001
–	–	−49.2 ± 5.2 *n* = 19	–	− 76.0 ± 9.8 *n* = 11	0.0245
–	–	–	− 137.6 ± 18.9 *n* = 15	−76.0 ± 9.8* *n* = 11	< 0.001

*, *p* < 0.05; One Way ANOVA test followed by Bonferroni

(−) indicates that the respective group is not part of the comparison on that row

## Discussion

It has been shown that the elevation in basal glycemia is capable of promoting an increase in plasma TNF-α concentration, even in healthy non-diabetic individuals. In experiments performed in cell culture, hyperglycemic medium stimulates inflammatory signaling pathways that activate nuclear factor-κB (NF-κB) [32–34]. This may promote the transcription of TNF-α and its insertion into the endocrine/paracrine cycle signaling linked to TNF-α release, followed by new activation of NF-κB [35]. Li et al. [36] showed in cell cultures that hyperglycemic medium induces the expression of the metalloproteinase ADAM-17 which is consistent with the ensuing elevation in TNF-α serum concentration. There are also clinical findings that associate pro-and anti-inflammatory cytokine elevation in the blood serum with the induction and persistence of DNP in diabetic patients [37–39]. Ortmann and Chattopadhyay [40] highlighted the importance of TNF-α as an additional pathogen in the development of diabetic neuropathy. These authors showed increased immuno-reactivity for TNF-α in histological sections of the DRG, dorsal horn of the spinal cord, sciatic nerve and paw skin of rats that developed hyperalgesia [41]. Several studies have correlated the elevation in plasma TNF-α concentration with alteration of the expression and/or function of voltage-dependent Na^+^ channels, critical elements in the establishment of neuronal excitability. In turn, this may be reflected, at least in part, in the reduction of the threshold for activation of the peripheral nerves, thus promoting the establishment of chronic neuropathic pain [42–46]. Thus, our data are in accordance with previous findings, since diabetic rats, with sustained hyperglycemia, exhibited both hyperalgesia and elevated TNF-α serum concentration levels.

Hyperalgesia to mechanical stimuli has been extensively reported in STZ-induced diabetic rats [47–50], and the data represented in Fig. 1 are in agreement with the literature. Like others [51–56], we observed an age-dependent increase in mechanical thresholds in control rats, whereas STZ injected rats showed a slight decrease, overall consistent with the development of diabetic neuropathic pain. In diabetic rats with hyperalgesia, DRG neurons are known to exhibit increased action potential frequency in response to sustained suprathreshold mechanical stimulation [47, 57, 58] and increased spontaneous activity [59]. Both effects are thought to contribute to the development of pain [43] and are related to the activity of voltage-activated Na^+^ channels. Among these Na^+^ channels, the Na_V_1.7 isoform has been associated with a crucial role in the development of the DNP. Na_V_1.7 channels are robustly expressed in the cell bodies of virtually all neurons that act as nociceptive fibers Aδ and C [19, 60]. They are also present in both peripheral and central termini, with expression in the intraepidermal nerve fibers within the skin and dorsal root horn surface lamina, the region of greatest synaptic connectivity between primary and secondary nociceptive neurons [25]. Nav1.7 expression is increased in diabetic rats [11, 20, 61] and this effect has been linked to TNF-α expression in the DRG of these animals [61]. Based on this and in the work of Tamura et al. [16], we investigated how exposure of dissociated DRG neurons to relevant TNF-α concentrations may affect their Na^+^ currents.

Our results showed that TNF-α induces an increase of both TTXs and TTXr current density, which contributes to the overall increase in total Na^+^ current. Ding and colleagues reported a TNF-α mediated increase in Nav1.6 expression in rat DRG neurons [62], whereas Chen et al. [63] observed no change in the expression of the Na^+^ channel isoforms Na_V_1.1, 1.2, 1.3 or 1.6 in response to 8 h exposure to a TNF-α concentration of 1000 pg/ml. On the other hand, Na_V_1.7 was shown to increase its expression after only 6 h exposure to the same concentration of TNF-α [16]. Although other groups reported differences in total, TTXs or TTXr currents after a shorter duration of TNF-α exposure, this can be explained by the notion that these authors used much higher TNF-α concentrations [28, 64, 65]. It is important to note that Na_V_1.7 is the main Na^+^ channel isoform expressed in Aδ and C fibers [66, 67], and hence it is thus possible that our observed changes in the TTXs Na^+^ current occurred are due to an increase in Na_V_1.7 expression. Further experimentation will be needed to attribute the expression of specific Nav isoforms confirm to our whole cell recordings.

We also observed a functional effect of TNF-α on the activation gating of the TTXs current component, an effect not seen with the TTXr Na^+^ current. A possible mechanism by which TNF-α alters the gating of TTXs channels may be through the ERK1 and ERK2 kinase phosphorylation and perhaps via p38 MAPK, both of which are activated by TNF receptors [21, 23, 68]. The latter mechanism has been reported to augment Na_V_1.8 single channel conductance [28, 59, 63], which would match the increased current density of the TTXr current. Increases in Nav1.8 and Nav1.9 expression in transgenic mice with elevated TNF-α levels have also been reported [31]. Indeed, we note that the TTXr Na^+^ current observed in our experiments may be carried by Na_V_1.8 channels, since the pipette solution does not contain CsF (or even F^−^), necessary for the recording of the Na^+^ current conducted by the Na_V_1.9 isoform [69]. As noted above, a detailed dissection at the molecular level will be needed to validate such a possibility.

The mechanism by which TNF-α induces the increase in Na_V_1.7 channel expression has not yet been clarified. Recently, Dustrude et al. [70] demonstrated that Na_V_1.7 expression in the cell membrane can be modulated by the cytoplasmic protein CRMP2. This protein is highly expressed in neurons and oligodendrocytes of the central nervous system [47]. During central nervous system development, this protein also performs regulatory and structural functions related to cytoskeletal dynamics, vesicle traffic and synaptic activity, while its functions in the adult brain are still being elucidated. In addition, CRMP2 has been correlated with various neuropathological or psychiatric conditions, including Alzheimer’s disease and schizophrenia [71]. CRMP2 can be phosphorylated at several sites, SUMOylated, undergo addition of a β-N-acetyl-D-glucosamine group and be oxidized [72]. Among these, SUMOylation is a covalently reversible binding process between small ubiquitin-like modifying proteins (SUMO1, 2 or 3) and the substrate. According to the work of Dustrude et al. [70], inhibition of CRMP2 SUMOylation in DRG neurons reduces Na_V_1.7 channels trafficking from the nucleus to the cellular membrane surface. Moreover, depletion of CRMP2 leads to a reduction in the Na^+^ current density via Na_V_1.7. In our hands, DRG neurons expressing the CRMP2-K374A protein showed reduced total Na^+^ current density when compared to both the control group neurons and neurons expressing the CRMP2-WT protein, in agreement with the work of Dustrude et al. [70]. Nonetheless, TNF-α potentiated TTXs currents in the presence of either WT or mutant CRMP2, indicating that TNF-α effects occur independently of CRMP2. Further work will be required to define the precise cell signaling pathways that underlie the action of TNF-α on Na^+^ channels in sensory afferents.

In conclusion, TNF-α, at a serum concentration similar to that measured in STZ-induced diabetic rats, is capable of modulating Na^+^ current in dissociated DRG neurons after 6 h exposure. Although this effect is independent of SUMOylation of CRMP2, the TNF-α mediated enhancement of Na^+^ channel expression could potentially be exploited for therapeutic intervention into diabetic pain.

## Data Availability

The data used in our study are available from the authors on reasonable request.

## Abbreviations

CRMP2: collapsin response mediator protein 2
DNP: diabetic neuropathy pain
DRG: Dorsal root ganglia
Nav: voltage dependent sodium channel
PDN: peripheral diabetic neuropathy
STZ: Streptozotocin
TNF-α: tumor necrosis factor alpha
TTX: tetrodotoxin
TTXr: tetrodotoxin resistant
TTXs: tetrodotoxin sensitive
